# Concurrent and long-term associations between the endometrial microbiota and endometrial transcriptome in postpartum dairy cows

**DOI:** 10.1186/s12864-019-5797-8

**Published:** 2019-05-22

**Authors:** Stephen G. Moore, Aaron C. Ericsson, Susanta K. Behura, William R. Lamberson, Timothy J. Evans, Matthew S. McCabe, Scott E. Poock, Matthew C. Lucy

**Affiliations:** 1 Division of Animal Sciences, University of Missouri, Columbia, MO 65211 USA; 2 Animal and Grassland Research and Innovation Centre, Teagasc Moorepark, Fermoy, Co. Cork, P61 P302 Ireland; 3 Department of Veterinary Pathobiology, University of Missouri, Columbia, 65211 USA; 4 University of Missouri Metagenomics Center, University of Missouri, Columbia, 65201 USA; 5 Informatics Institute, University of Missouri, Columbia, MO 65211 USA; 6 Animal and Grassland Research and Innovation Centre, Teagasc Grange, Dunsany, Co. Meath, C15 PW93 Ireland; 7 College of Veterinary Medicine, University of Missouri, Columbia, 65211 USA; 8 Division of Animal Sciences, 160 Animal Science Research Center, 920 East Campus Drive, University of Missouri, Columbia, MO 65211 USA

**Keywords:** Uterus, Bacteria, Gene expression, Ovarian cyclicity, Progesterone, Microbiome, Principal component analysis, Limma, RNAseq, 16S rRNA

## Abstract

**Background:**

Fertility in dairy cows depends on ovarian cyclicity and on uterine involution. Ovarian cyclicity and uterine involution are delayed when there is uterine dysbiosis (overgrowth of pathogenic bacteria). Fertility in dairy cows may involve a mechanism through which the uterine microbiota affects ovarian cyclicity as well as the transcriptome of the endometrium within the involuting uterus. The hypothesis was that the transcriptome of the endometrium in postpartum cows would be associated with the cyclicity status of the cow as well as the microbiota during uterine involution. The endometrium of first lactation dairy cows was sampled at 1, 5, and 9 weeks postpartum. All cows were allowed to return to cyclicity without intervention until week 5 and treated with an ovulation synchronization protocol so that sampling at week 9 was on day 13 of the estrous cycle. The endometrial microbiota was measured by 16S rRNA gene sequencing and principal component analysis. The endometrial transcriptome was measured by mRNA sequencing, differential gene expression analysis, and Ingenuity Pathway Analysis.

**Results:**

The endometrial microbiota changed from week 1 to week 5 but the week 5 and week 9 microbiota were similar. The endometrial transcriptome differed for cows that were either cycling or not cycling at week 5 and cyclicity status depended in part on the endometrial microbiota. Compared with cows cycling at week 5, there were large changes in the transcriptome of cows that progressed from non-cycling at week 5 to cycling at week 9. There was evidence for concurrent and longer-term associations between the endometrial microbiota and transcriptome. The week 1 endometrial microbiota had the greatest effect on the subsequent endometrial transcriptome and this effect was greatest at week 5 and diminished by week 9.

**Conclusions:**

The cumulative response of the endometrial transcriptome to the microbiota represented the combination of past microbial exposure and current microbial exposure. The endometrial transcriptome in postpartum cows, therefore, depended on the immediate and longer-term effects of the uterine microbiota that acted directly on the uterus. There may also be an indirect mechanism through which the microbiome affects the transcriptome through the restoration of ovarian cyclicity postpartum.

**Electronic supplementary material:**

The online version of this article (10.1186/s12864-019-5797-8) contains supplementary material, which is available to authorized users.

## Background

Female fertility in dairy and beef cattle is an important component of agricultural productivity and farm profitability worldwide [1]. For cows after calving, conception rate after insemination progressively improves for the first 60 days postpartum. The improvement in conception rate over time depends on the resumption of ovarian cyclicity and the involution of the uterus. Uterine involution occurs coincident with the proliferation of bacteria in the uterus during the first week after calving. The presence of bacteria in the uterus is normal in postpartum cows [2–4] and there are bacteria from the external environment and also bacteria remnant from the previous pregnancy that contribute to the microbiota postpartum [5, 6]. Overgrowth of pathogenic bacteria (dysbiosis) may occur leading to metritis; a disease defined by a foul smelling uterine discharge, fever, and loss of appetite within the first week postpartum.

Ten to 20% of dairy cows fail to begin cycling by the start of the breeding period [7, 8]. Once they begin cycling and are inseminated, these late-cycling cows will have decreased conception rates and increased pregnancy loss [7–9]. Cycles before breeding, therefore, may condition the uterus and conceptus through a mechanism involving estradiol and progesterone (P4) from the cyclic ovary [10].

The objective of this study was to explore the relationships between the endometrial microbiota and the endometrial transcriptome in cows that were or were not cycling by 5 weeks postpartum. We performed 16S rRNA gene sequencing of endometrial bacteria at 1, 5 and 9 weeks postpartum and tested for both concurrent and long-term associations of the microbiota with the endometrial transcriptome. The hypothesis was that the endometrial microbiota would be associated with the differential gene expression within the transcriptome of the postpartum endometrium and that effects of cyclicity on the endometrium would also be identified. There was a major effect of cyclicity status on the endometrial transcriptome. We also found evidence for both a concurrent and long-term association of the endometrial microbiota with the endometrial transcriptome and also associations of the microbiome with the resumption of ovarian cyclicity. This later observation may implicate an indirect mechanism through which the endometrial microbiota can mediate endometrial function through a pathway that involves restoration of ovarian cyclicity postpartum. The endometrial transcriptome in postpartum cows, therefore, depends on the immediate and longer-term effects of the uterine microbiota that act either directly or through an indirect mechanism that involves the restoration of ovarian cyclicity postpartum.

## Results

### Composition of the endometrial microbiota

*Average number of 16S rRNA sequence reads.* The average number of 16S rRNA sequence reads generated from the endometrial microbiota of the postpartum cows was greater for week 1 compared with either week 5 (*P =* 0.01) or week 9 (*P =* 0.03) and was similar for week 5 and week 9 (*P =* 0.25). There was a week 5 ovarian cyclicity status x week postpartum interaction (*P =* 0.01) for the average number of 16S rRNA sequence reads. Compared with the week 5 cycling (CycW5) cows, the week 5 non-cycling (NoCycW5) cows had a greater number of 16S rRNA sequence reads on week 1 [5302 (1926–14,598) vs. 1134 (384–3351) reads; LSM with 95% CI; *P =* 0.04]. The CycW5 and NoCycW5 cows had a similar number of reads on week 5 [211 (77–582) vs. 848 (287–2509) reads; *P =* 0.07] and week 9 [496 (180–1366) vs. 1449 (490–4283) reads; *P =* 0.15].

### Relative abundance of operational taxonomic units (OTU)

**Fig. 1:**
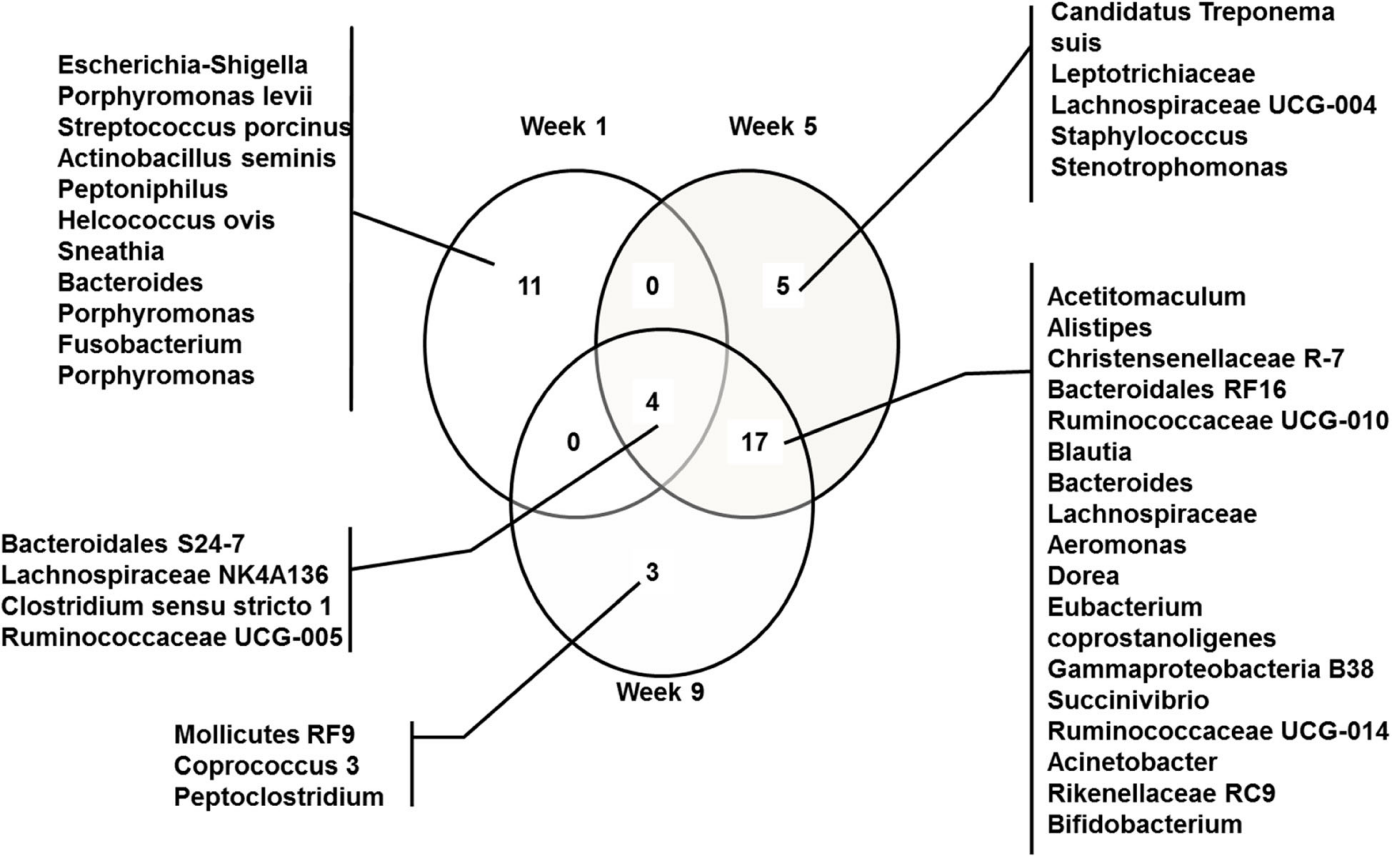
Venn diagram of the endometrial microbiota with a relative abundance greater than 1% at week 1, 5, and 9 postpartum in lactating dairy cows. Eleven, 5, and 3 operational taxonomic units (OTU) with a mean relative abundance of 56, 7, and 5% were unique at week 1, 5, and 9, respectively. Seventeen OTU were unique at week 5 and 9, and their combined relative abundance increased from 10.9% on week 5 to 20.0% on week 9 (*P =* 0.004). Four OTU were present on each week and their combined relative abundance was greater on week 5 (19.8%) and 9 (20.3%) compared with week 1 (8.8%; *P =* 0.004)

The relative abundance of each OTU is provided in Additional file 1 Table S1. Only OTU with an average relative abundance of at least 1% were retained for analysis. Eleven, 5, and 3 OTU with a mean relative abundance of 56, 7, and 5% were unique to week 1, 5, and 9, respectively (Fig. 1). Seventeen OTU were unique to week 5 and 9, and their combined relative abundance increased from 10.9% on week 5 to 20.0% on week 9 (*P =* 0.004). Four OTU (*Bacteroidales S24–7*, *Lachnospiraceae NK4A136*, *Clostridium* sensu stricto *1*, and *Ruminococcaceae UCG-005*) were present on each week and their combined relative abundance was greater on week 5 (19.8%) and 9 (20.3%) compared with week 1 (8.8%; *P =* 0.004).

**Fig. 2:**
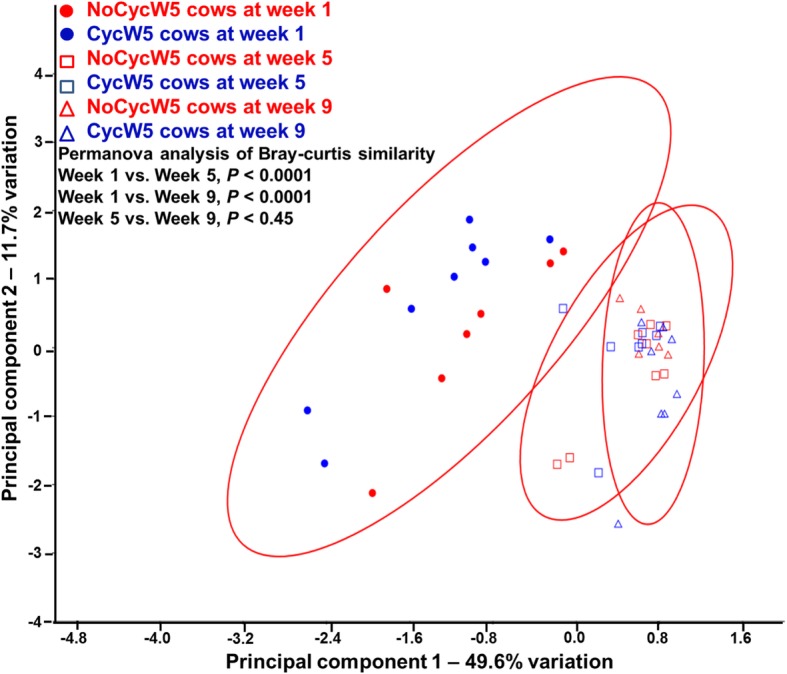
Plot of principal components PC1 vs. PC2 generated from the endometrial microbiota at week 1, 5, and 9 postpartum. Separation of the week 1 endometrial microbiota from both week 5 and 9 is evident. Permanova analysis indicated that the Bray-Curtis similarity index (a measure of microbial similarity) was significantly different between week 1 and 5 (*P =* 0.0001) and between week 1 and 9 (*P* = 0.0001) and similar between week 5 and 9 (*P* = 0.45). Each 95% concentration ellipse estimates a region where 95% of population points are expected to fall

When principal components (PC) were generated for the postpartum microbiota on week 1, 5, and 9, a plot of PC1 vs. PC2 illustrated the separation of the week 1 endometrial microbiota from both week 5 and 9 (Fig. 2). Permanova analysis indicated that the Bray-Curtis similarity index (a measure of microbial similarity) was significantly different between week 1 and week 5 (*P =* 0.0001) and week 1 and 9 (*P =* 0.0001). The Permanova analysis did not detect a difference between week 5 and 9 microbiota (*P =* 0.45).

The CycW5 and NoCycW5 cows differed with respect to the relative abundance of OTU at certain sampling times. The relative abundance of *Actinobacillus seminis* on week 1 was greater in the NoCycW5 cows compared with the CycW5 cows (29.9% vs. 13.6%, Wilcoxon *P =* 0.049). The relative abundance of *Gammaproteobacteria_B38* on week 5 (0.2% vs. 1.9%, Wilcoxon *P =* 0.004) and 9 (0.7% vs. 2.6%, Wilcoxon *P =* 0.005) was greater in NoCycW5 cows compared with the CycW5 cows. The relative abundance of *Aeromonas* on week 9 was greater in the NoCycW5 cows compared with the CycW5 cows (8.6% vs. 0.6%, Wilcoxon *P =* 0.003).

**Fig. 3:**
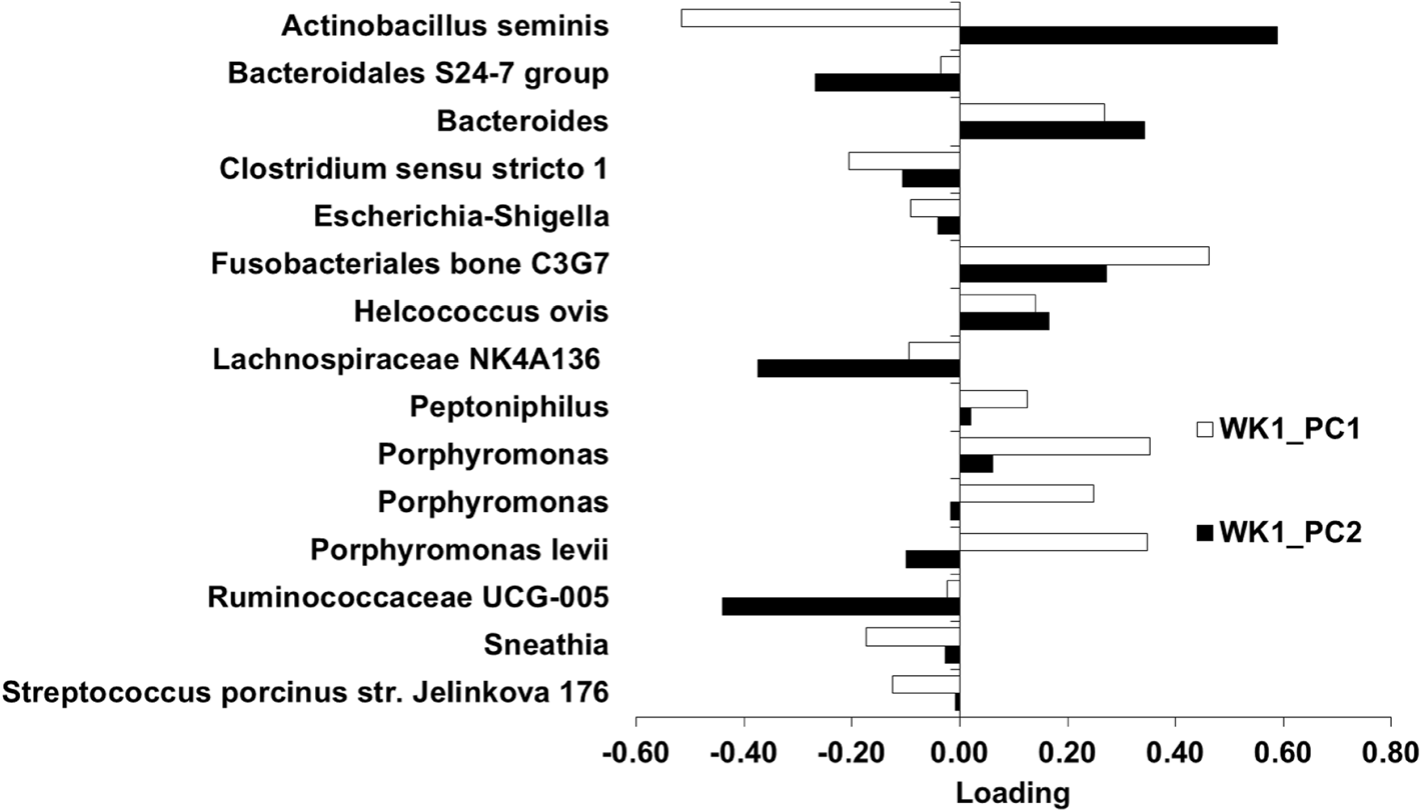
Plot of loadings from the endometrial microbiota principal components WK1_PC1 and WK1_PC2

**Fig. 4:**
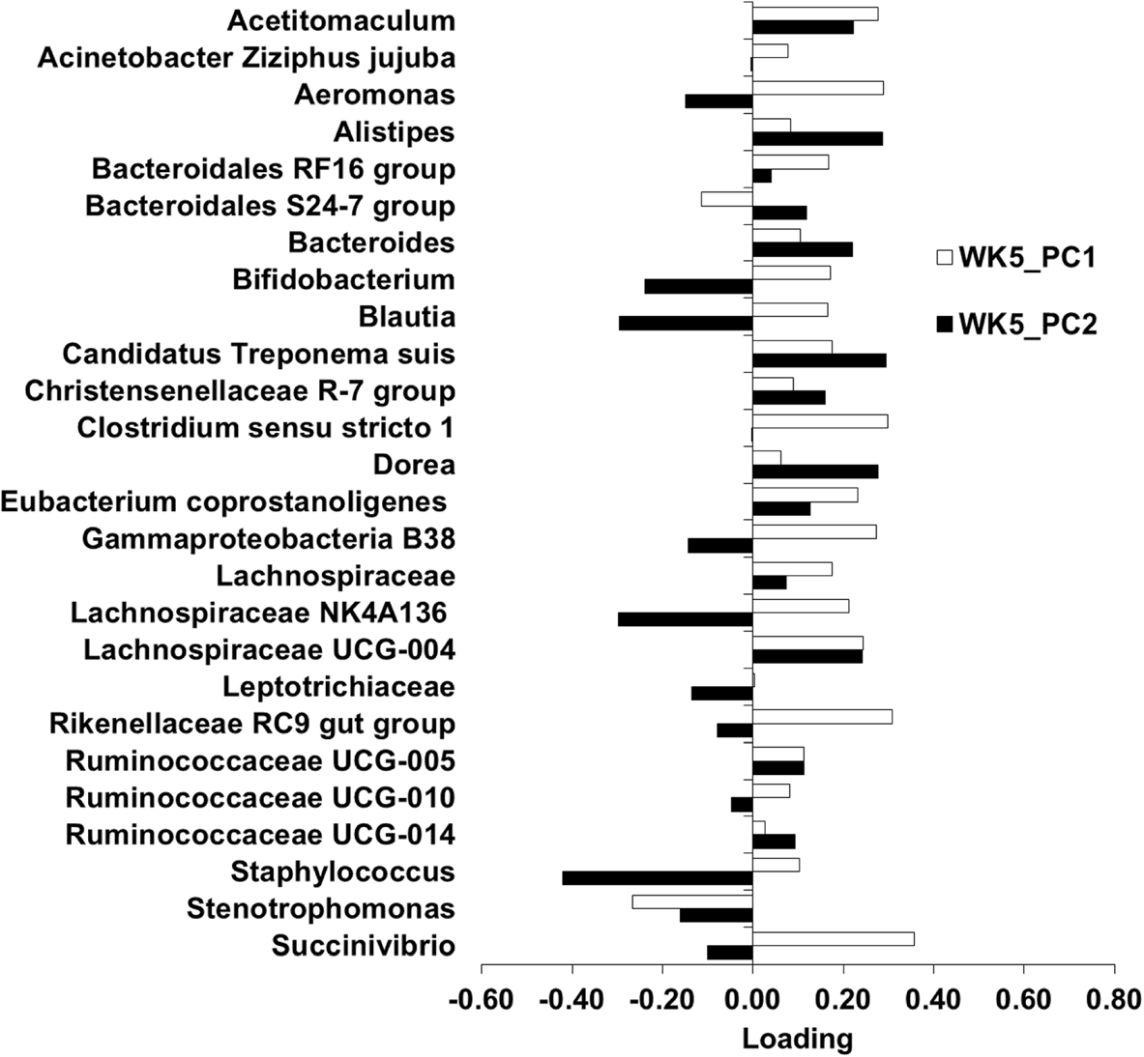
Plot of loadings from the endometrial microbiota principal components WK5_PC1 and WK5_PC2

**Fig. 5:**
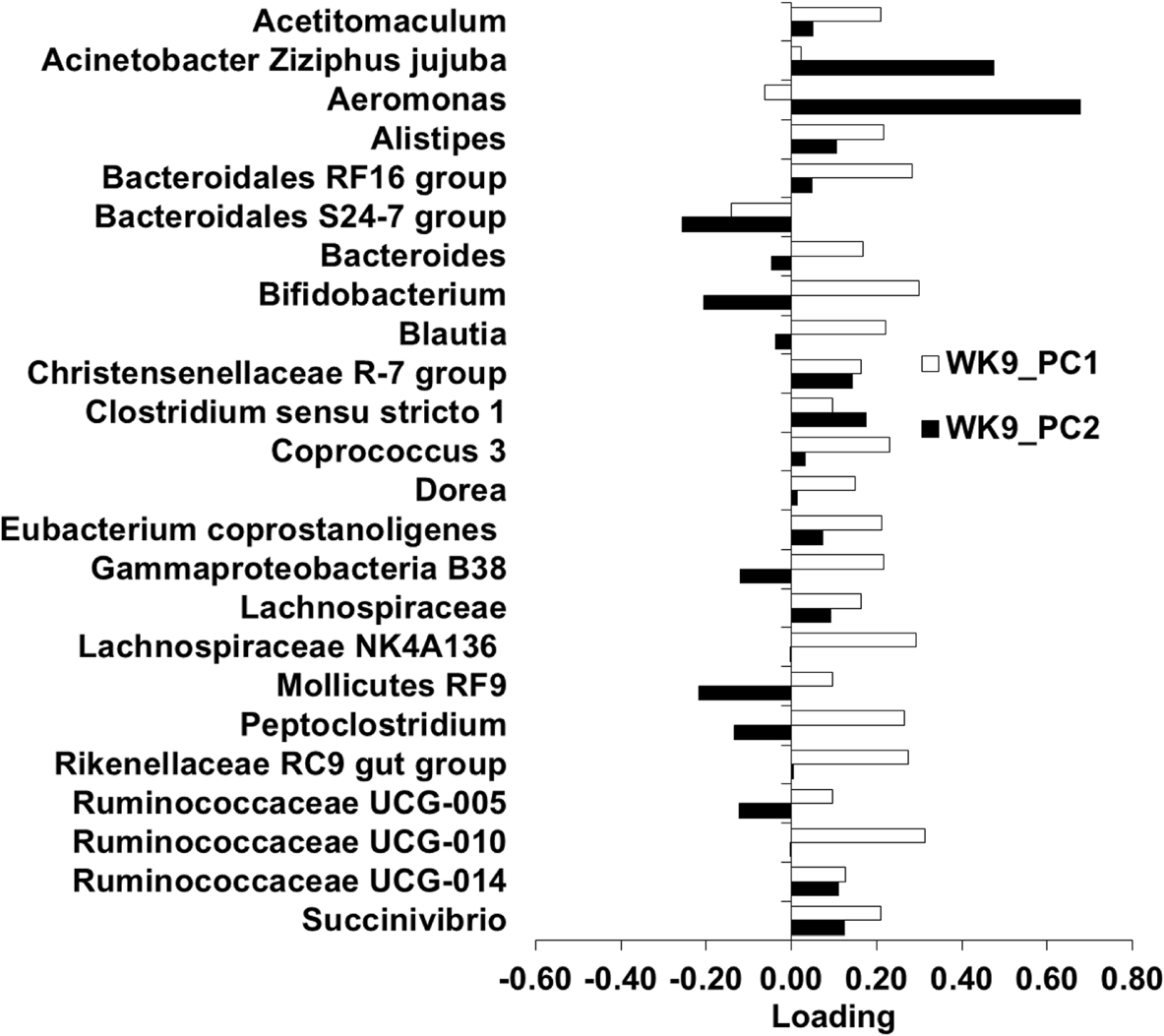
Plot of loadings from the endometrial microbiota principal components WK9_PC1 and WK9_PC2

Subsequent analyses were performed on the microbiota for each individual week. The PC1 and PC2 on each week were the focus of the current study because they explained most of the variation in the microbiota. The PC1 and PC2 for week 1 (WK1_PC1 microbiota, WK1_PC2 microbiota), week 5 (WK5_PC1 microbiota, W5_PC2 microbiota), and week 9 (WK9_PC1 microbiota, and WK9_PC2 microbiota) accounted for 27.5, 16.9, 26.0, 11.4, 38.4, and 13.9% of the variation within each week, respectively. The loading plot for each PC was examined to identify the most influential OTU (Figs. 3, 4, 5). The top loadings for WK1_PC1 microbiota were *Fusobacteriales bone C3G7* and *Porphyromonas* (Fig. 3), and for WK1_PC2 microbiota were *Actinobacillus seminis*, *Bacteroides*, *Fusobacteriales bone C3G7*, and *Helcococcus ovis* (Fig. 3). No predominant loadings were identified for the WK5_PC1 microbiota, WK5_PC2 microbiota (Fig. 4), and WK9_PC1 microbiota (Fig. 5). The top loadings for WK9_PC2 microbiota were *Aeromonas* and *Acinetobacter Ziziphus jujuba* (Fig. 5).

### Effect of ovarian cyclicity and the week 1 and week 5 microbiota on the endometrial transcriptome at 5 weeks postpartum

**Fig. 6:**
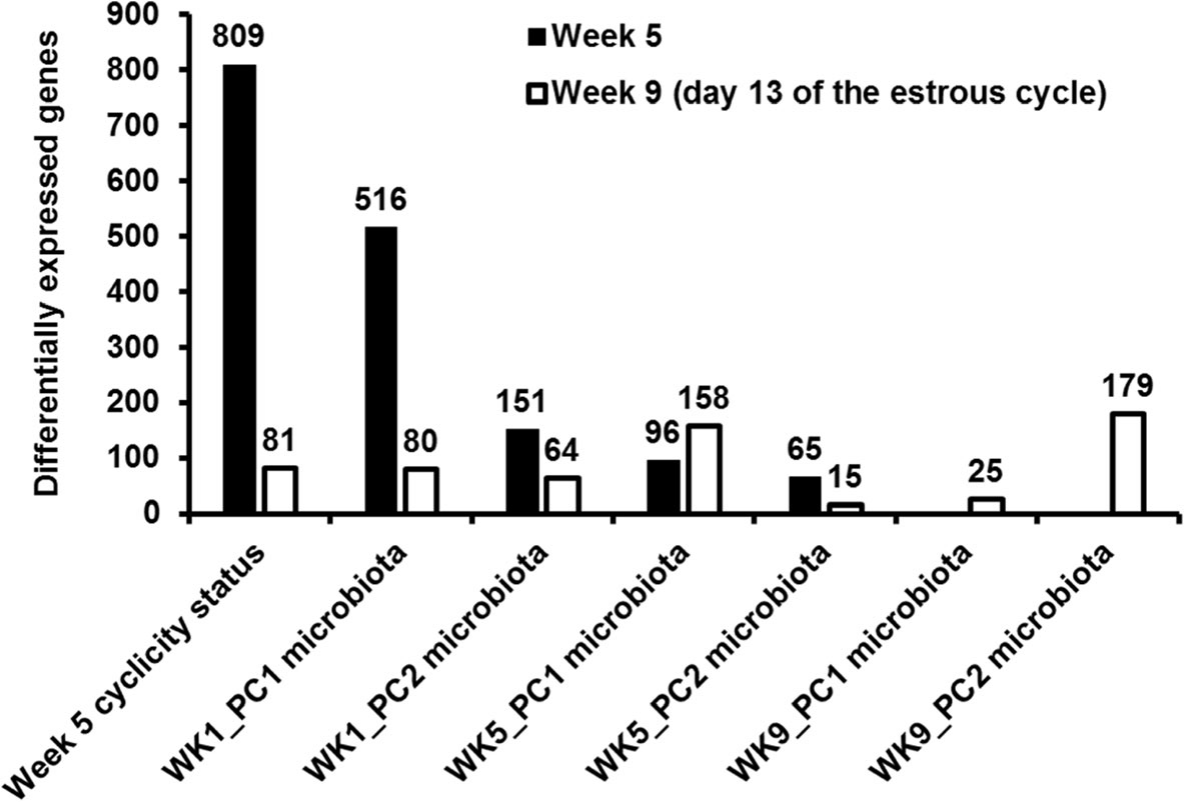
Numbers of differentially expressed genes in endometrium of lactating dairy cows at weeks 5 and 9 postpartum associated with ovarian cyclicity status (cycling at week 5, non-cycling at week 5), and the endometrial microbiota present at week 1 (WK1_PC1, WK1_PC2), week 5 (WK5_PC1, WK5_PC2), and week 9 (WK9_PC1, WK9_PC2)

There were 809 genes differentially expressed between the CycW5 cows and the NoCycW5 cows on week 5 (BH *P* ≤ 0.05; Fig. 6; Additional file 1: Table S2). Based on the Ingenuity Pathway Analysis (IPA) of the differentially expressed genes (DEG), the CycW5 cows had a down-regulation of the canonical pathway EIF2 signaling (i.e., protein synthesis) and a down-regulation of the biological functions cell death, necrosis, astrocytosis, and liver lesion compared with the NoCycW5 cows (Table 1). There was an up-regulation in the CycW5 cows of the biological functions transport of molecule, transport of lipid, oxidation of lipid, quantity of carbohydrate, cell-to-cell contact, fusion of cells, formation of muscle, and head and neck cancer (Table 1). Target molecules of differentially expressed genes in the CycW5 cows were involved in increased molecule transport of glutamine (*SLC38A1*, BH *P =* 0.007), arginine, lysine, ornithine (*SLC7A4*, BH *P =* 0.009 and *SLC25A9*, BH *P =* 0.02), zinc (*SLC30A5*, BH *P =* 0.04 and *SLC30A6*, BH *P =* 0.04), copper (*SLC31A2* BH *P =* 0.02), thiamine (*SLC19A2*, BH *P =* 0.005), and sialic acid (*SLC17A5*, BH *P =* 0.04).

**Table 1:** Ingenuity Pathway Analysis of endometrial genes differentially expressed at week 5

Pathway or Biological Function	Z-score	*P*-value
Cyclic vs non-cyclic (*n* = 809 DEG)		
Canonical Pathway - Decreased		
EIF2 signaling (i.e., protein synthesis)		6.16E-10
Biological Functions - Increased		
Transport of molecule	2.809	5.01E-05
Transport of lipid	2.753	7.24E-03
Oxidation of lipid	2.325	5.03E-03
Quantity of carbohydrate	2.057	1.72E-04
Cell-cell contact	2.295	5.25E-03
Fusion of cells	2.266	2.02E-03
Formation of muscle	2.209	3.63E-03
Head and neck cancer	2.4	6.69E-03
Biological Functions - Decreased		
Cell death	−2.193	2.11E-03
Necrosis	− 2.493	2.45E-03
Astrocytosis	−2.353	3.53E-03
Liver lesion	−2.452	1.71E-04
Week 1 microbiota (WK1_PC1; n = 516 DEG)		
Biological functions - Increased		
Thoracic hypoplasia	2.44	1.73E-05
Cardiac lesion	2.423	8.65E-03
Fibrosis of heart	2.241	6.66E-03
Biological functions - Decreased		
Organization of cytoplasm	−2.383	1.32E-03
Organization of cytoskeleton	−2.383	1.41E-03
Proliferation of neuroblastoma cell lines	−2.412	6.95E-03
Formation of muscle cells	−2.607	4.07E-04
Formation of muscle	−2.902	2.44E-04
Week 5 microbiota (WK5_PC1; *n* = 96 DEG)		
Biological functions - Decreased		
Organismal death	−2.35	3.58E-03
Thoracic hypoplasia	−2	7.07E-03

There were 516 genes differentially expressed (BH *P ≤* 0.05; Fig. 6; Additional file 1: Table S3) on week 5 in response to the first PC derived from the microbiota present at week 1 (WK1_PC1 microbiota; Fig. 3). These DEG represent the association between the microbiota present on week 1 and the endometrial transcriptome present 4 weeks later (week 5). Based on the IPA, the WK1_PC1 microbiota was associated with a down-regulation of the biological functions organization of cytoplasm, organization of cytoskeleton, proliferation of neuroblastoma cell lines, formation of muscle cells, and formation of muscle, and an up-regulation of the biological functions thoracic hypoplasia, cardiac lesion, and fibrosis of heart (Table 1). There were 151 genes differentially expressed on week 5 in response to the second PC derived from the microbiota present at week 1 [WK1_PC2 microbiota (Fig. 3); BH *P ≤* 0.05; Additional file 1: Table S4]. The IPA did not predict significant activation of any pathways or biological processes.

There were 96 genes differentially expressed (BH *P ≤* 0.05; Fig. 6; Additional file 1: Table S5) in response to the first PC derived from the microbiota present at week 5 (WK5_PC1 microbiota; Fig. 6). These DEG represent the association between the microbiota present on week 5 and endometrial transcriptome at the same time. Based on the IPA, the WK5_PC1 microbiota was associated with down-regulation of the biological functions organismal death and thoracic hypoplasia on week 5. There were 65 genes differentially expressed (BH *P ≤* 0.05; Fig. 6; Additional file 1: Table S6) in the week 5 transcriptome in response to the second PC derived from the microbiota present at week 5 (WK5_PC2 microbiota; Fig. 4) but the IPA did not predict significant activation of any pathways or biological processes.

### Changes in the endometrial transcriptome from week 5 to week 9 (day 13 of the estrous cycle) for cows that were either cycling or non-cycling at week 5

**Fig. 7:**
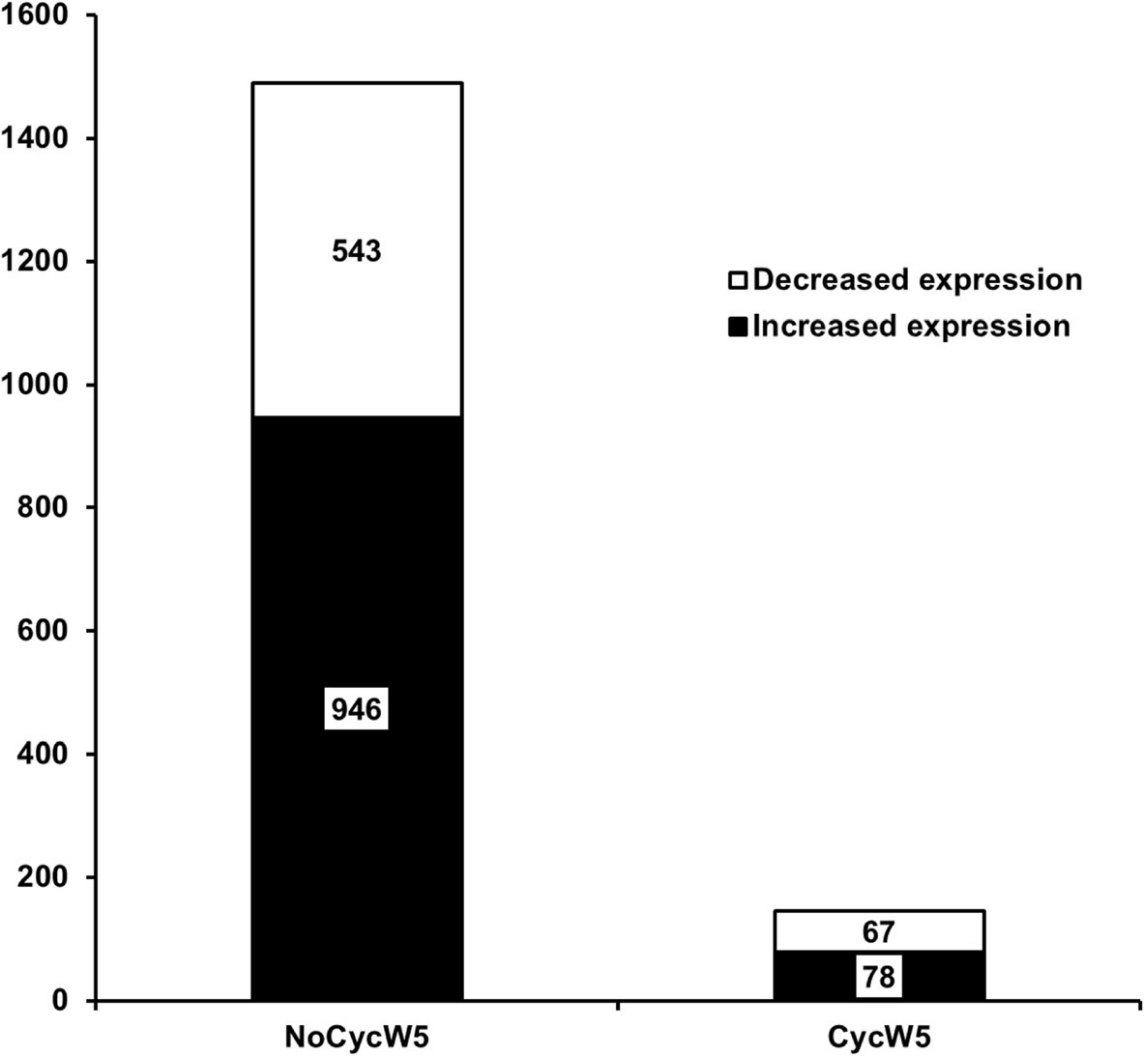
Numbers of differentially expressed genes in endometrium of lactating dairy cows from week 5 to 9 postpartum in cows cycling at week 5 and in cows non-cycling at week 5

**Fig. 8:**
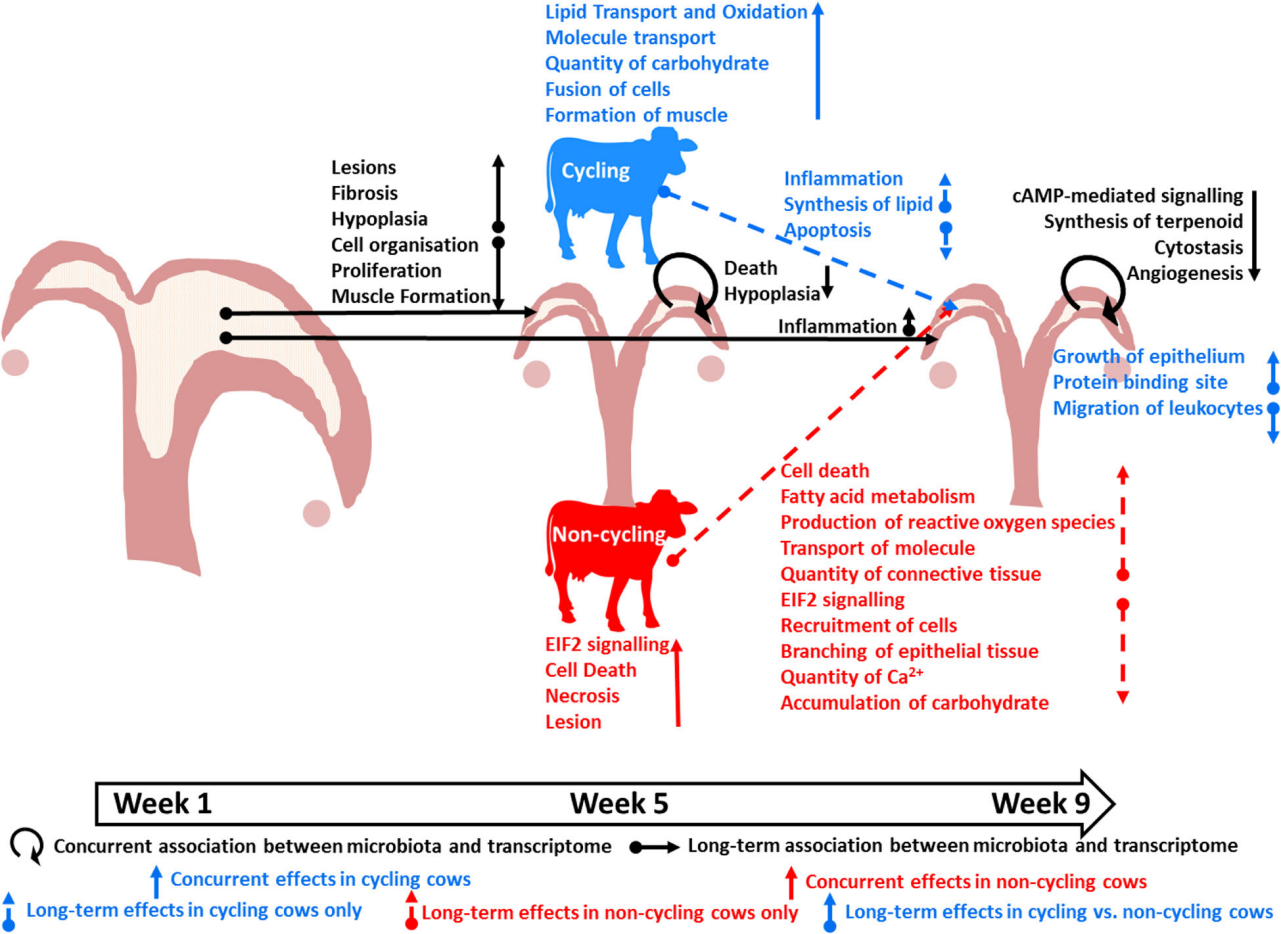
Schematic summary of the concurrent and long-term effects of ovarian cyclicity and the endometrial microbiota on the endometrial transcriptome

Temporal changes in the endometrial transcriptome between week 5 and week 9 were dependent on the ovarian cyclicity status at week 5 (Fig. 7). Cows that were non-cycling at week 5 (NoCycW5) were all cycling on day 13 of the estrous cycle when sampled at week 9. There were 1489 genes differentially expressed between week 5 and week 9 in the NoCycW5 cows (BH *P ≤* 0.05; Fig. 8; Additional file 1: Table S7). The IPA predicted a down-regulation of the canonical pathway EIF2 signaling, a down-regulation of biological functions recruitment of cells, branching of epithelial tissue, quantity of Ca^2+^, and accumulation of carbohydrate at week 9 compared with week 5 (Table 2). There was an up-regulation of the biological functions cell death of tumor cells, cancer, conversion of fatty acid, metabolism of membrane lipid derivative, production of reactive oxygen species, transport of molecule, and quantity of connective tissue at week 9 compared with week 5 in the NoCycW5 cows (Table 2).

**Table 2:** Ingenuity Pathway Analysis of endometrial genes differentially expressed between week 5 and week 9

Pathway or Biological Function	Z-score	P-value
Non-cyclic (week 5) to cyclic (week 9) (n = 1489 DEG)		
Canonical Pathway - Decreased		
EIF2 signaling (i.e., protein synthesis)		2.13E-25
Biological Functions - Increased		
Cell death of tumor cells	2.407	6.98E-05
Conversion of fatty acid	2.757	1.90E-05
Metabolism of membrane lipid derivative	2.745	9.74E-08
Production of reactive oxygen species	2.091	1.05E-05
Transport of molecule	2.365	4.63E-07
Quantity of connective tissue	2.325	5.08E-06
Cancer	2.257	1.82E-05
Biological Functions - Decreased		
Recruitment of cells	−2.211	4.52E-05
Branching of epithelial tissue	−2.081	5.96E-05
Quantity of Ca2+	−2.111	1.11E-05
Accumulation of carbohydrate	−2.44	2.36E-05
Cyclic (week 5) to cyclic (week 9) (n = 145 DEG)		
Biological Functions - Increased		
Inflammation of lung	2.187	1.09E-02
Synthesis of lipid	2.115	7.80E-03
Biological Functions - Decreased		
Apoptosis	−2.814	2.98E-03

Compared with the previous comparison, cows that were cycling at week 5 and week 9 had numerically fewer DEG between week 5 and 9 (*n* = 145; BH *P ≤* 0.05; Fig. 7; Additional file 1: Table S8). The IPA predicted a down-regulation of the biological function apoptosis and up-regulation of the biological function inflammation of lung and synthesis of lipid on week 9 compared with week 5 for cows that were cycling at week 5 in the CycW5 cows (Table 2).

Of the 1489 and 145 genes differentially expressed between week 5 and week 9 in the NoCycW5 cows and the CycW5 cows, respectively, there was an overlap of 39 genes that represented 3 and 27% of the respective gene lists. The fold change in gene expression in response to cyclicity status was highly correlated (R^2^ = 0.79; Additional file 2: Figure S1).

### Effect of week 5 ovarian cyclicity status and the week 1, week 5, and week 9 microbiota on the endometrial transcriptome at 9 weeks postpartum (day 13 of the estrous cycle)

The endometrial transcriptome on week 9 (d 13 of the estrous cycle) was affected by the cyclicity status of the cows at week 5 (CycW5 versus NoCycW5) as well as the microbiota present at week 1 (8 weeks earlier), week 5 (4 weeks earlier), and week 9 (present at the time of tissue collection) (Fig. 6).

There were 81 genes differentially expressed at week 9 between the CycW5 cows and the NoCycW5 cows (BH *P ≤* 0.05; Fig. 6; Additional file 1: Table S9). These DEG represented an association between previous ovarian cyclicity status at week 5 and the endometrial transcriptome at week 9 when all cows were on d 13 of the estrous cycle. The analysis differs from that presented in the previous section where gene expression fold changes from week 5 to week 9 were presented. The IPA predicted a down-regulation of the biological function migration of mononuclear leukocytes and an up-regulation of the biological functions growth of tumor, neoplasia of epithelial tissue, and binding of protein site at week 9 in the CycW5 cows compared with the NoCycW5 cows (Table 3). Of the 809 and 81 genes differentially expressed between the CycW5 cows and the NoCycW5 cows on week 5 and week 9, respectively, there was an overlap of 21 genes that represented 3 and 26% of the respective gene lists. The fold change in gene expression in response to cyclicity status was highly correlated (R^2^ = 0.97; Additional file 2: Figure S2).

**Table 3:** Ingenuity Pathway Analysis of endometrial genes differentially expressed at week 9

Pathway or Biological Function	Z-score	P-value
Cyclic vs non-cyclic (*n* = 81)		
Biological Functions - Increased		
Growth of tumor	2.618	2.30E-06
Neoplasia of epithelial tissue	2.425	2.89E-03
Binding of protein binding site	2.19	4.56E-03
Biological Functions - Decreased		
Migration of mononuclear leukocytes	− 2.219	4.57E-03
Week 1 microbiota (WK1_PC2; n = 64 DEG)		
Biological functions - Increased		
Inflammatory response	2.914	2.34E-07
Influx of myeloid cells	2.19	7.34E-07
Quantity of leukocytes	2.115	3.74E-08
Week 9 microbiota (WK9_PC2; n = 179 DEG)		
Canonical Pathway - Decreased		
cAMP-mediated signaling	−2.646	1.09E-03
Biological Functions - Increased		
Malignant solid tumor	2.007	4.23E-06
Biological Functions - Decreased		
Synthesis of terpenoid	−2.528	1.18E-02
Cytostasis	−2.16	1.23E-02
Angiogenesis	−2.155	7.16E-05

There were 64 genes that were differentially expressed (BH *P ≤* 0.05; Additional file 1: Table S10) in response to the WK1_PC2 microbiota (Fig. 6) on week 9. These DEG represent the association between the second PC of the week 1 endometrial microbiota and the endometrial transcriptome on week 9. The IPA predicted up-regulation of biological functions inflammatory response, influx of myeloid cells, and quantity of leukocytes (Table 3). A variety of up-stream regulators for the observed gene expression signature associated with WK1_PC2 microbiota were predicted based on the IPA (Table 4). These upstream regulators included response to bacterial products [lipopolysaccharide, endotoxin B, peptidoglycan, and 5-O-mycolyl-β-Araf-(1 → 2)-5-O-mycolyl-α-Araf-(1 → 1′)-glycerol (designated as Mma_DMAG)], interleukins (IL1, IL1A, IL1B, IL6, IL10RA, IL17A), tumor necrosis factor, and toll-like receptors (TLR3, TLR4, TLR5, TLR7, TLR9).

**Table 4:** Predicted upstream regulators of endometrial genes differentially expressed at week 9 (day 13 of the estrous cycle) in response to WK1_PC2

Upstream regulator	Z-score^a^	P-value	Differentially expressed genes (week 9)^c^
Bacterial products			
Mma_DMAG^b^	2.45	5.67E-08	CCL20, CXCL2, NFKBIZ, SDC4, SOD2, TNIP3
Enterotoxin B	2.41	2.88E-08	CSF3, CTLA4, CXCL13, CXCL2, IL12B, IL17A
Lipopolysaccharide	3.63	9.88E-10	CCL20, CHI3L1, CSF3, CXCL13, CXCL2, IFNW1, IL12B, IL17A, IL22, IL36A, LAMB1, LCN2, LYN, NFKBIZ, PC, PFKFB3, RAB20, SDC4, SOD2, TNIP3
Peptidoglycan	2.39	2.65E-07	CCL20, CSF3, CXCL2, IL12B, IL17A, SOD2
Interleukins			
IL1	2.28	1.40E-06	CCL20, CHI3L1, CSF3, IL17A, IL22, LAMB1, SDC4, SOD2
IL1A	2.56	6.29E-08	ALDH1A3, CCL20, CXCL2, IL12B, IL17A, LCN2, NFKBIZ, SOD2
IL1B	3.39	1.30E-09	CCL20, CHI3L1, CSF3, CXCL13, CXCL2, GDF15, IL12B, IL17A, IL22, IL36A, LCN2, NFKBIZ, SDC4, SLC22A4, SOD2
IL6	2.75	5.56E-05	CCL20, CLEC10A, CXCL13, CXCL2, IL12B, IL17A, IL22, LCN2, SOD2
IL10RA	−2.00	9.07E-04	CTLA4, IL12B, IL17A, IL18BP, LCN2
IL17A	2.18	5.87E-08	CCL20, CSF3, CXCL13, CXCL2, IL17A, IL22, LCN2, NFKBIZ
Tumor necrosis factor	3.62	2.72E-12	AATK, ALDH1A3, CCL20, CHI3L1, CHI3L2, CSF3, CTLA4, CXCL13, CXCL2, GDF15, IL12B, IL17A, IL18BP, IL22, IL36A, ITGA10, LCN2, LYN, NFKBIZ, PC, SDC4, SLC22A4, SOD2
Toll-like receptors			
TLR3	2.91	8.70E-09	CCL20, CSF3, CXCL2, IFNW1, IL12B, IL36A, LCN2, NFKBIZ, PFKFB3
TLR4	2.88	1.04E-09	CCL20, CSF3, CXCL2, DUOX2, IFNW1, IL12B, IL17A, IL18BP, LCN2, NFKBIZ, SOD2
TLR5	2.18	2.81E-08	CCL20, CSF3, CXCL2, IL12B, IL17A
TLR7	2.39	1.52E-06	CCL20, CXCL13, CXCL2, IFNW1, IL12B, SOD2
TLR9	2.57	1.07E-06	CSF3, IFNW1, IL12B, IL36A, LCN2, NFKBIZ, PFKFB3

^a^positive z-score = increased activation; negative z-score = decreased activation

^b^5-O-mycolyl-beta-araf-(1 → 2)-5-O-mycolyl-alpha-araf-(1 → 1′)-glycerol

^c^See Additional file 1: Table S10 for gene description

There were 179 genes differentially expressed (BH *P ≤* 0.05; Additional file 1: Table S11) in response to the WK9_PC2 microbiota (Fig. 6). These DEG represent the association between the second PC of the week 9 endometrial microbiota and the endometrial transcriptome on week 9. The IPA predicted down-regulation of the canonical pathway cAMP-mediated signaling and the biological functions synthesis of terpenoid, angiogenesis, and cytostasis and up-regulation of the biological function malignant solid tumor (Table 3).

There were 80, 158, 15, and 25 genes differentially expressed (BH *P ≤* 0.05) on week 9 in response to WK1_PC1 microbiota (Additional file 1: Table S12), WK5_PC1 microbiota (Additional file 1: Table S13), WK5_PC2 microbiota (Additional file 1: Table S14), and WK9_PC1 microbiota (Additional file 1: Table S15), respectively (Fig. 6). The IPA did not predict significant activation of any pathways or biological processes for these PC.

Of the 516 and 80 genes differentially expressed on week 5 and week 9 in response to WK1_PC1, there was an overlap of 27 genes that represented 5 and 34% of the respective gene lists. The fold change in gene expression in response to WK1_PC1 microbiota at week 5 for these overlapping genes was highly correlated (R^2^ = 0.97; Additional file 2: Figure S3). A similar analyses identified a minimal overlap (< 5 genes) when the effect of WK1_PC2 microbiota, WK5_PC1 microbiota, and WK5_PC2 microbiota on differential gene expression at week 5 and week 9 was assessed.

### Endometrial histology, endocrine and physical characteristics of the study population

The number of endometrial inflammatory cells was not associated with week postpartum, week 5 cyclicity status, or the endometrial microbiota (all *P* > 0.1). Lymphocytic foci were absent at week 1 but were present in samples collected at week 5 and 9. Their number decreased by 50% from week 5 to 9 (4.4 ± 0.7 vs. 2.2 ± 0.7 foci, *P* = 0.02) and was not associated with the endometrial microbiota or week 5 cyclicity status (all *P* > 0.1). None of the cows enrolled in the study were diagnosed with clinical disease based on the measurement of body temperature or the presence of fetid discharge within the vagina (metricheck diagnosis). Milk production was associated with endometrial microbiota 1 week postpartum and with the week 5 cyclicity status. Energy-corrected milk production tended to be greater during the first 20 weeks postpartum in the CycW5 cows compared with the NoCycW5 cows (26.2 vs. 23.4 kg day^− 1^, SEM = 1.0 kg day^− 1^; *P* = 0.08) and increased significantly (5.58 ± 2.34 kg day^− 1^ unit^− 1^, *P =* 0.05) in response to WK1_PC2 microbiota with a predicted increase of 6.25 kg day^− 1^ for the range in WK1_PC2 microbiota (− 0.49 to 0.63) observed. No effects of week 5 ovarian cyclicity status or the endometrial microbiota on body weight, body condition score, vaginal mucus score, rectal temperature, or circulating concentrations of beta-hydroxy butyrate, glucose, IGF1, fatty acids, or progesterone were detected (all *P* > 0.1).

## Discussion

The primary objective of the current study was to test for associations between the endometrial microbiota and the transcriptome in early postpartum cows. The hypothesis was that the endometrial microbiota would be associated with the transcriptome of the endometrium. The capacity of the microbiota to alter the endometrial transcriptome could partly influence fertility performance in lactating dairy cows.

The microbiota within the endometrial samples at week 1 was highly abundant and these data are consistent with the published literature [2, 11–14]. There was a decrease in the number of 16S rRNA sequence reads from week 1 to week 5 and week 9 that appeared to demonstrate the resolution of bacterial infection over time. The composition of the microbiota also changed from week 1 to weeks 5 and 9 but the similarity between week 5 and 9 indicates that a stable microbiota is established within 5 weeks after calving and remains unchanged until at least 9 weeks postpartum (Figs. 1 and 2). *Fusobacteriales bone C3G7*, *Porphyromonas*, *Actinobacillus seminis*, *Bacteroide*s, and *Helcococus ovis* had the strongest loadings in the microbiota PC at WK1 but were not detected at week 5 or at week 9. With the exception of *Actinobacillus seminis,* the other predominant bacteria are frequently reported to be more abundant in the uterus of cows that develop metritis compared with healthy cows [2–4, 11–14].

Of the 35 cows enrolled in this study, data from seven cows that had resumed ovarian cyclicity at week 5 (CycW5) and eight cows that had not resumed ovarian cyclicity at week 5 (NoCycW5) were retained for analysis. Mobilization of adipose reserves in the postpartum cow shifts the metabolism and endocrinology of the cow to negatively affect the return to ovarian cyclicity through mechanisms acting at the level of the hypothalamus and pituitary [15]. Systemic bacterial toxins can negatively affect feed intake and exacerbate negative energy balance and weight loss postpartum. Bacterial products also act directly on the developing follicle to affect its capacity to respond to gonadotropins and ovulate [16–18]. The dry matter intake of the cows was not recorded in this study but blood metabolic indicators were similar in the CycW5 and the NoCycW5 cows. Although ovarian cyclicity status had no effect on the endometrial microbiota principal components, the number of 16S rRNA gene sequence reads and the relative abundance of *Actinobacillus seminis* were greater on week 1 in the NoCycW5 cows compared with the CycW5 cows. The IPA of the week 5 transcriptome data indicated that endometrium of the NoCycW5 cows had more tissue damage (necrosis, cell death, astrocytosis, and lesion up-regulated) and was less advanced with respect to tissue repair (formation of muscle, fusion of cells, and cell-cell contact down-regulated; Table 1, Fig. 8). Systemic products of the incompletely involuted uterus may have delayed the onset of cyclicity through an effect on the hypothalamic-pituitary-gonadal axis, as previously outlined [19–22]. This latter scenario is an utero-centric view toward early postpartum cyclicity in the bovine that includes the progression of uterine involution which in part is dictated by the uterine microbiota [16–18]. In this scenario, delayed cyclicity is caused by signals arriving from the uterus that are in part dictated by the microbiota.

Given the well-documented effects of P4 on the endometrium [23–26], the microbiota cannot be viewed separately from cyclicity when studying the postpartum endometrial transcriptome. To address this challenge, the endometrial transcriptome was tested using a complete statistical model. Ovarian cyclicity status had a large effect on endometrial gene expression on week 5 (809 DEG) and the effect of cyclicity status on the number of DEG was numerically greater than the effect of the microbiota (PC) on the number of DEG (Fig. 6). Based on the IPA, the DEG on week 5 between the CycW5 cows and the NoCycW5 cows were found within the canonical pathway EIF2 signaling (protein synthesis) which was decreased in the CycW5 cows (Table 1, Fig. 8). Similarly, there was a large number of DEG between week 5 and week 9 in the NoCycW5 cows, and the EIF2 signaling pathway was decreased on week 9 (Table 2, Fig. 8). The NoCycW5 cows that were not cycling at week 5 and were cycling at week 9 and had undergone a large downward change in the expression of genes involved in protein synthesis. Progesterone modulates the synthesis and secretion of specific proteins in endometrium [27, 28] but an effect of P4 on the sum of all transcription has not been demonstrated to our knowledge. The global up-regulation of endometrial protein synthesis in the NoCycW5 cows at week 5 may also reflect the ongoing tissue repair and regeneration in the endometrium.

Progesterone is a known inhibitor of cell death and this function was supported by greater mRNA abundance of apoptosis inhibitor *BCL2* and lesser mRNA abundance of tumor necrosis factor receptors (*TNFRSF1A* and *TNFRSF25*) and cell death inducing p53 target 1 (*CDIP1*) in the CycW5 cows compared with the NoCycW5 cows on week 5 (Additional file 1: Table S2). Greater mRNA abundance of myosin (*MYO1A*, *MYO1B*, *MYOF*, *TPM1*, *MYMR9*, *MYOT*), actin (*ABLIM1*, *CTTN*, and *MACF1*), and collagen (*DSC3*, *COL4A3*, and *COL4A4*) related genes in the CycW5 cows compared with the NoCycW5 cows on week 5 (Additional file 1: Table S2) may have contributed to increased cell support, cytokinesis, and transport, and to actin-myosin interactions in the development of cytoskeleton and ECM in the endometrium of the CycW5 cows [29].

The CycW5 cows had an up-regulation of the biological functions lipid transport and oxidation, molecule transport, and quantity of carbohydrate compared with the NoCycW5 cows on week 5 (Table 1, Fig. 8). Squalene synthase (*FDFT1*) and lanosterol synthase (*LSS*) comprise two of the three enzymes involved in synthesis of lanosterol (cholesterol precursor) from farnesyl diphosphate. mRNA abundance of both genes was increased in the CycW5 cows on week 5 (Additional file 1: Table S2). The CycWk5 cows also had greater mRNA abundance of *ESR2*, the prostaglandin inactivator *HPGD*, and transporters with a preference for the prostaglandin precursor arachidonic acid (*ACSL3* and *ACSL4*) on week 5. These data support the role of P4 in the accumulation of endometrial lipid droplets in preparation of prostaglandin synthesis while concurrently inhibiting prostaglandin synthesis as discussed by Silvia et al. 1991 [30].

The first PC of the week 1 microbiota (WK1_PC1) had the second largest effect on gene expression at week 5 (*n* = 516 DEG) after ovarian cyclicity status (Fig. 6). The second PC of the week 1 microbiota (WK1_PC2) had a numerically smaller effect at week 5 (*n* = 151 DEG) than the WK1_PC1 microbiota and this is perhaps explained by the fact that the PC2 by definition accounts for less of the variation than the PC1. For both WK1_PC1 microbiota and WK1_PC2 microbiota the decrease in the number of DEG at week 9 compared with week 5 (Fig. 6) indicates that some of the effects of the early postpartum microbiota are not permanent. This may indicate that bacteria associated with the transcriptome are gradually cleared from the uterus or that uterine inflammation associated with the early postpartum microbiota gradually subsides.

Ingenuity Pathway Analysis indicated lesion, fibrosis, and hypoplasia associated with the WK1_PC1 microbiota effect on the endometrium at week 5 (Table 1, Fig. 8). This result is consistent with the observation that unresolved inflammation causes fibrosis through excessive deposition of ECM. At the same time, the IPA detected decreased organization, proliferation, and formation of muscle cells (Table 1, Fig. 8). The collective interpretation is that the microbiota present at week 1 is associated with unique patterns of gene expression at week 5. In addition to their associations with metritis, many of the OTU with strong loadings in WK1_PC1 are also associated with specific pathologies that are also supportive of the Ingenuity Pathway Analysis of the week 5 endometrial transcriptome. *Fusobacteria* and *Porphyromonas* reduce wound healing in human oral epithelium via increased cell apoptosis and compromised cell migration and cell proliferation [31] and are also associated with papillomatous digital dermatitis in cattle [32]. *Porphyromonas levii*, specifically, is associated with bovine necrotic vulvovaginitis [33] and may also reduce the phagocytic capacity of polymorphonuclear neutrophils [34]. There was also evidence that the OTU associated with WK1_PC1 microbiota were affecting the week 5 transcriptome through a direct effect on transcription factor expression. Transcription factor DEG associated with WK1_PC1 fell broadly into three categories that included steroid receptors and associated molecules (*AR*, *ESR1*, *GMEB2*, *MED1*, *RXRB*), immune function and inflammation (*LEF1*, *LYL1*, *NFATC3*, *NR1H2*) and cell growth including embryonic development and cell differentiation (*CREB1*, *FOXN2*, *GLI3*, *HIF1A*, *PRDM5*, *RBPJ*, *SMAD5*, *SOX15*, *SOX18*, *STAG1*, *TCF4*, *TCF25*, *TFDP2*; Additional file 1: Table S3). The microbiota of PC2 from week 1 was also associated with transcription factor expression at week 5. The DEG list was shorter and specifically focused on tissue differentiation (*FOXA1*, *FOXC2*, *GATA5*, *HAND1*, *HEY2*, *MEOX2*, *NKX2–2*, *POU2F1*, and *SOX2*; Additional file 1: Table S4). Collectively, the DEG at week 5 possessed transcription factors involved in cell growth and differentiation that are responding to the microbiota present at week 1.

A major conclusion from this work, therefore, was that, transcription factor expression at week 5 was in response to the microbiota present at week 1. The observed effect of the week 1 microbiota on the week 5 endometrial transcriptome indicates that endometrial tissue at week 5 has not fully recovered from the exposure to the microbiota present at week 1. This may indicate an effect of the week 1 microbiota on the progression of uterine involution, the population of endometrial cell types and specific patterns of gene expression in the week 5 endometrium. The capacity for the week 1 microbiota to dictate transcription on week 5 within regenerative cells arising from stem cells could be explained by mechanisms similar to that described by Naik et al. (2017) where there is inflammatory memory in skin epithelial stem cells [35]. This memory is created when an inflammatory event creates an open chromatin configuration around specific genes that is maintained for up to 180 days [35].

The sum total of week 5 DEG in response to the week 1 microbiota (PC1 + PC2) was 667. The sum total of week 5 DEG in response to the week 5 microbiota (PC1 + PC2; present in the uterus at the time the tissue was collected; Fig. 6) was appreciably less (161 DEG). The microbiota present 4 weeks prior, therefore, had a larger effect on week 5 DEG than the microbiota present at the time of tissue sampling. The IPA identified organismal death and thoracic hypoplasia in the week 5 endometrium response to the week 5 microbiota (PC1; Table 1, Fig. 8). An association between the microbiota and the expression of transcription factors in the endometrium at week 5 was also detected. This was true for both WK5_PC1 and WK5_PC2. There was some overlap with the transcription factors affected by the week 1 PC’s and this may be explained by overlapping microorganisms between week 1 and week 5. There were also week 5 transcription factors involved in cellular differentiation (*DLX5*, *HOXC6*, *TBX15*, and *TEAD1;* Additional file 1: Table S5 and Additional file 1: Table S6) associated with the week 5 microbiota that were not associated with the week 1 microbiota. Mechanisms through which a resident microbiota can impact local gene transcription typically involve the production of bacterial products that bind TLR to invoke an inflammatory response [36]. Bacteria also secrete products that can bind mammalian hormone receptors to initiate cellular responses. Cohen et al. (2017) [37] demonstrated that commensal bacteria of the gut produced molecules that bind to human G-protein coupled receptors to trigger endocrine responses. Similarly, commensal bacteria of the endometrium may control endometrial gene expression.

One of the primary objectives of this research was to determine if the early postpartum microbiota could affect transcription at the time of first insemination (approximately 9 weeks postpartum). There was a large decrease in the number of DEG in response to the WK1_PC1 microbiota from week 5 (*n* = 516 DEG) to week 9 (*n* = 80 DEG; Fig. 6). Similarly, the number of DEG responding to WK1_PC2 microbiota decreased from week 5 (*n* = 151) to week 9 (*n* = 64; Fig. 6). The effects of the week 1 microbiota on the total number of DEG, therefore, diminished with time. There was some overlap between the DEG in response to WK1_PC2 microbiota at both week 5 to week 9 with 27 genes shared between the two gene lists (Additional file 1: Table S3). This number of genes presented about 5% of the DEG at week 5 that remained DEG at week 9. The IPA did not detect an effect of the WK1_PC1 microbiota on gene expression at week 9 (Additional file 1: Table S12). There was a large effect, however, of the WK1_PC2 microbiota on DEG at week 9 (Table 3, Additional file 1: Table S10). The IPA clearly identified an effect of the WK1_PC2 microbiota on immune and inflammatory response within the endometrium at week 9 (inflammatory response, influx of myeloid cells, and quantity of leukocytes; Table 3, Fig. 8). Predicted upstream regulators included several bacterial products, a variety of interleukins, TNF, and several TLR (Table 4). The biological functions identified and their upstream regulators were all highly significant. These data are remarkable in that they identify a large effect of the early postpartum microbiota specifically on the inflammatory processes at week 9. The upstream regulators include bacterial products (Mma_DMAG, enterotoxin B, LPS, and peptidoglycan) potentially arising from the microbiota at week 1, TLR with the capacity to respond to bacterial products and interleukins arising from the immune cells involved in inflammation (IL1, IL1A, IL1B, IL6, and IL17A). The list of genes differentially expressed in response to WK1_PC2 was compared with the list of endometrial genes differentially expressed after in vitro treatment with LPS, as reported by Oguejiofor et al. [18]. With the exception of *ST6GAL2*, 10 of the 11 genes (*ALDH1A3*, *CCL20*, *CSF3*, *IL36A*, *KCNB1*, *LYN*, *MAB21L3*, *PFKFB3*, *PGLYRP3*) common to both lists were up-regulated in response to WK1_PC2 and to LPS treatment (R^2^ = 0.22). Such concordance was not observed between the other gene lists. The mechanisms for this long-term effect may be similar to those described for the effect of the week 1 microbiota on the week 5 transcriptome. Specifically, the week 1 microbiota may be affecting the progression of uterine involution, the population of endometrial cell types (in this case the number of resident immune cells), or specific patterns of gene expression at week 9. Operational taxonomic units with strong loadings in WK1_PC2 are associated with inflammatory disease in other species that support the IPA of the week 9 endometrial transcriptome. *Actinobacillus seminis* had a strong positive loading in WK1_PC2 and is associated with epididymitis (inflammation of the epididymis) in rams and abortion in ewes [38, 39]. In contrast, *Bacteroidales S24–7* and *Lachnospiraceae* had strong negative loadings in WK1_PC2 and are depleted after the onset of colitis (gut inflammation) [40]. Although there were effects of the week 5 microbiota (WK5_PC1, *n* = 158 DEG; WK5_PC2, n = 15 DEG) on the transcriptome (Fig. 6), the IPA did not discover specific biological functions associated with these DEG. Major effects on the functionality of the endometrial transcriptome at breeding therefore arise from bacteria of the early postpartum uterus. The impact of the later postpartum microbiota may be less.

There was a large effect of previous cyclicity status on DEG at week 9. The NoCycW5 cows that transitioned from non-cycling (week 5) to cycling (week 9) had nearly 1500 DEG (Fig. 7). The EIF2 signaling pathway was greatly decreased at week 9 (Table 2). There was an increase in a variety of biological functions associated with metabolism and transport and a decrease in recruitment and branching of cells (Table 2, Fig. 8). The number of DEG between week 5 and week 9 for the CycW5 cows was one-tenth in number (*n* = 145 DEG) when compared between week 5 and week 9 for the NoCycW5 cows (*n* = 1489 DEG; Fig. 7). The large number of DEG in cows that began cycling between week 5 and 9 compared with those that were cycling at both week 5 and 9 demonstrated the large effect that P4 has on endometrial gene expression.

A novel and important finding from the study was evidence of temporal effects of week 5 ovarian cyclicity status on the endometrial transcriptome 4 weeks later on day 13 of the estrous cycle (Table 3). The effects of week 5 cyclicity status on the endometrial transcriptome on day 13 of the estrous cycle were less pronounced than 4 weeks previous (Table 1) but sufficient to support significant temporal effects on the endometrial transcriptome. Greater fertility in cows that have an earlier return to ovarian cyclicity after calving is well documented [7–9] but the mechanisms have not been well studied. Transcriptome analysis of conceptus cells recovered 15 days after AI from cows that were previously cyclic vs. non-cyclic indicated lesser cellular stress and less apoptosis and autophagy [10]. Results from the current study also implicate mechanisms involving down-regulation of inflammation and up-regulation of epithelial cell growth in the week 9 endometrium of the CycW5 cows compared with the NoCycW5 cows.

The current study extends to week 9 postpartum the period when bacteria have been previously reported in the bovine uterus [2–4, 13]. These results indicate that the bovine uterus is not sterile at the time of first insemination. The endometrium was biopsied on day 13 of the estrous cycle because it represents a critical time point for embryo development in cattle, coinciding with the initiation of conceptus elongation and the secretion of IFNT for maternal recognition of pregnancy [41]. The WK9_PC1 microbiota was associated with 25 endometrial DEG (Fig. 6). Although the WK9_PC2 microbiota explained less of the biological variation in the microbiota, there were a greater number of endometrial DEG associated with the WK9_PC2 microbiota (*n* = 179). The IPA indicated increased tumor growth and decreased angiogenesis and synthesis of terpenoids (also known as isoprenoids) in response to WK9_PC2 microbiota (Table 3, Fig. 8). Isoprenoids are derived from the mevalonate pathway that leads to cholesterol synthesis. It is possible that reduced isoprenoid synthesis represents a host-associated mechanism to inhibit bacterial growth [42] or also an effect of the microbiota on the endometrium [43, 44]. Treatment with isoprenoids (farnesyl pyrophosphate and geranylgeranyl pyrophosphate) and inhibition of the mevalonate pathway (i.e., likely increasing isoprenoid concentrations) is known to increase endometrial stromal cell tolerance to bacterial pyolysin [43, 44]. Endometrial cAMP-mediated signaling was also decreased in response to WK9_PC2 microbiota (Table 2; Fig. 8) implicating a role for bacteria-derived molecules that bind to G-protein coupled receptors to trigger such endocrine responses, as described by Cohen et al. (2017) [37] in the human gut.

## Conclusions

The current study describes changes in the endometrial microbiota and transcriptome from week 1 to 9 postpartum in cows that were either cycling (CycW5) or not cycling (NoCycW5) at week 5 postpartum. The relationship between the uterine microbiota and endometrial transcriptome was explored. There were associations between uterine microbiota at week 1 and cyclicity at week 5. Bacterial species in the uterus early postpartum, therefore, may control the timing of first ovulation perhaps through systemic effects of products arising from uterine bacteria. Cyclicity status on week 5 affected endometrial gene expression at week 9 when all cows were cycling and on day 13 of the cycle. The capacity of the microbiota to affect cyclicity at week 5, therefore, may have implications for the functionality of the uterus later postpartum at week 9 when cows are inseminated. There was evidence for concurrent, short-term and longer-term associations between the endometrial microbiota and transcriptome (Fig. 8). The week 1 microbiota had the largest effect on the subsequent endometrial transcriptome and this effect diminished over time (wk 5 to wk. 9). Transcription factors represented one of the major classes of genes affected by the microbiota were discovered. Changes in transcription factor expression in response to the microbiota, therefore, may explain the transcriptome profile observed within endometrium. The uterine microbiota at week 1 was associated with an inflammatory response within the uterus at week 9 that included bacterial products, interleukins, TNF and toll-like receptors as upstream regulators. The association between the week 1 microbiota and the week 9 transcriptome differed from the association between the week 9 microbiota and the week 9 transcriptome (concurrent association). The cumulative effect of the microbiota on endometrial function at the time of breeding, therefore, represents the combined effects of past microbial exposure that may permanently imprint the transcriptome of cells and current microbial exposure that affects endometrial function in real time. The current study represents a preamble to future work where causation and mechanism will be investigated.

## Methods

### Animal management and sample collection

**Fig. 9:**
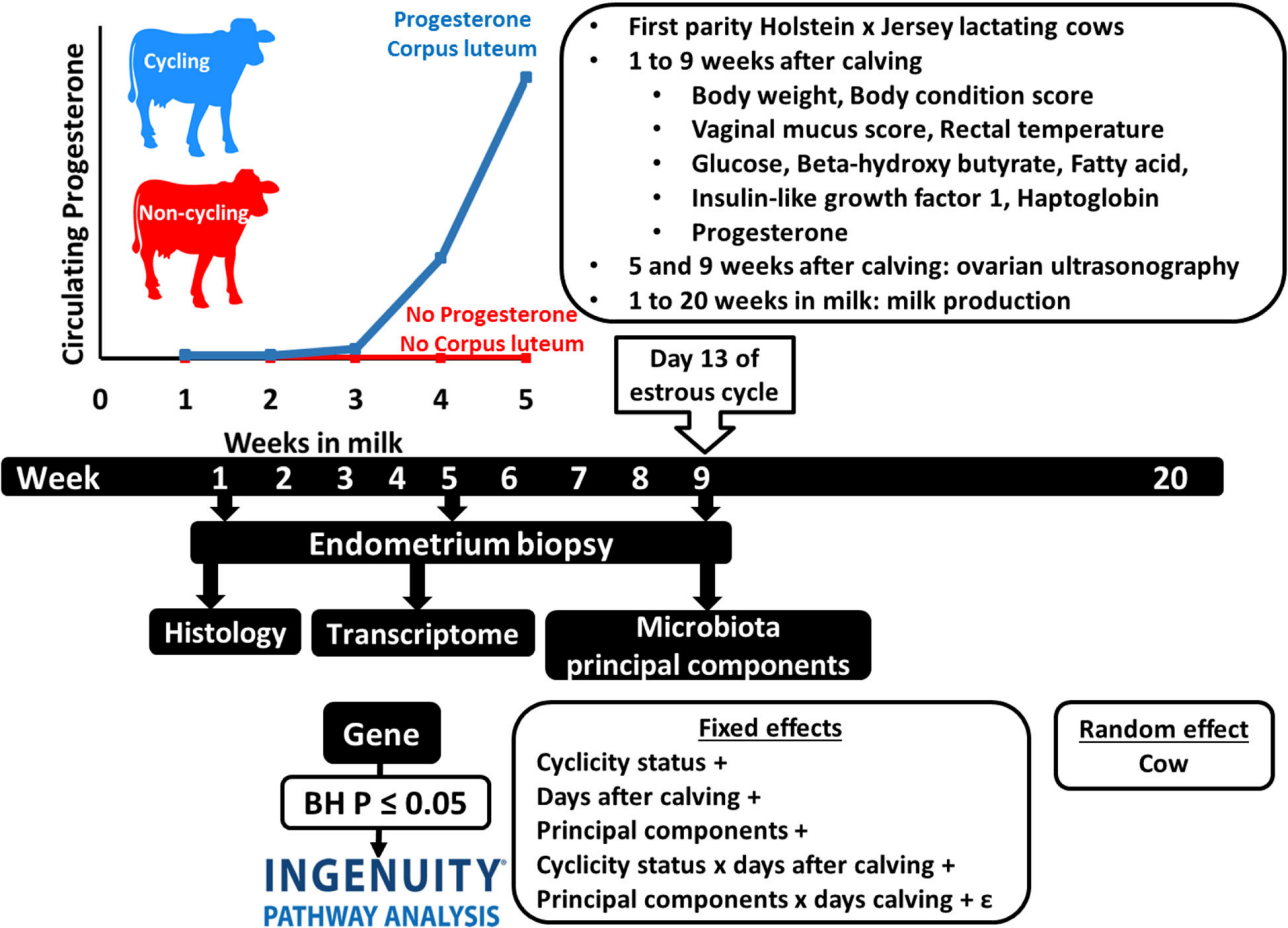
Overview of the experimental design and data analysis

An overview of the experimental design and data analysis is provided in Fig. 9. Thirty-five first lactation dairy cows of Holstein x Jersey admixture that calved during February 2016 on the University of Missouri Foremost Dairy Farm were enrolled. Cows calved in a dry lot barn and were managed in a freestall barn for the remainder of lactation. A total-mixed ration consisting of maize silage, soybean hulls, alfalfa hay, maize grain, and brewer’s grains was fed once daily and cows were milked twice daily at 12 h intervals. Milk yield was recorded at each milking using electronic milk meters (GEA United States, Columbia, MD). Milk composition (fat, protein, and somatic cell count) was determined monthly by flow cytometry and Fourier transformed infrared spectroscopy (Milkoscan/Fossomatic; Foss North America, Eden Prairie, MN) at Mid-South Dairy Records, Springfield, MO. Body weight, body condition score, rectal temperature, and vaginal mucus score were recorded and blood samples were collected immediately after the morning milking at 1 (7 ± 1 d), 2 (14 ± 1 d), 3 (21 ± 1 d), 4 (28 ± 1 d), 5 (35 ± 1 d), 6 (42 ± 1 d), 7 (49 ± 1 d), 8 (56 ± 1 d), and 9 (63 ± 1 d) weeks postpartum. Body condition score was assessed using the 1 to 5 scale in 0.25 increments [45]. For vaginal mucus scoring, the vulva and perineal area were sanitized with antimicrobial solution (2% chlorhexidine gluconate; VetOne, ID) and vaginal discharge was collected with a metricheck device (Simcro, Hamilton, New Zealand). Each vaginal discharge sample was scored as previously described [46]: 0 = clear and translucent mucus; 1 = mucus containing flecks of white or off-white pus; 2 = < 50% white or off-white mucopurulent material; or 3 = ≥50% white or off-white mucopurulent material. Diseased individuals were not specifically targeted in the present study and none of the animals were diagnosed with uterine disease based on rectal temperature or the presence of fetid uterine discharge. Blood samples were collected via coccygeal venipuncture into a Monoject tube containing EDTA (K3; Covidien, Minneapolis, MN) and placed on ice until centrifugation at 1500×g for 15 min at 4 °C; the plasma was then aspirated and stored at − 20 °C.

Beginning at 40 d postpartum, each cow was enrolled on an ovulation synchronization protocol so that 9 weeks postpartum coincided with day 13 of the estrous cycle. Each cow was administered a 100 μg i.m. injection of gonadorelin hydrochloride (GnRH; Factrel; Zoetis, New York, NY) and a controlled internal drug release (CIDR) insert containing 1.38 g of progesterone (P4; Eazi-Breed CIDR Cattle Insert; Zoetis). The CIDR insert was removed at 47 d postpartum and an i.m. injection of PGF_2α_ containing 25 mg of dinoprost tromethamine (Lutalyse; Zoetis) was administered. Each cow was administered a second i.m. injection of GnRH 56 h later.

Three samples of endometrium were collected from each cow on each day of sampling during 1, 5, and 9 weeks postpartum. At 1 week postpartum, a double-guarded plastic sheath was guided transcervically to the previously gravid uterine horn and endometrium was biopsied with a Wolf 8384.12 biopsy tool (Richard Wolf GmbH, Knittlingen, Germany). As the cervix was not dilated at 5 and 9 weeks postpartum, a standard stainless steel artificial insemination (AI) pipette fitted with a plastic AI sheath and a plastic coverall was guided transcervically to the uterine horn. Endometrial biopsies were collected ipsilateral to the CL when present and ipsilateral to the largest follicle when a CL was absent using a Wolf 8380.011 biopsy tool. On each day of sampling, the first and second biopsies were placed immediately in sterile tubes, snap-frozen in liquid nitrogen, and stored at − 80 °C until DNA and RNA extraction. The third biopsy was immediately fixed in 10% buffered formalin until histological analysis. Biopsy tools were cleaned, washed in antimicrobial solution (1:10 2% chlorhexidine gluconate), and rinsed with sterile filtered PBS before each biopsy.

After completion of the study, cows continued their lactation and were managed using standard operating procedures for the University of Missouri dairy farm.

### 16S rRNA gene sequencing and data processing

A manual precipitation protocol was used for DNA extraction [47]. Library construction and sequencing were performed by the University of Missouri DNA Core. A Qubit dsDNA BR Assay (Life Technologies, Carlsbad, CA) was used to determine DNA concentration. Samples were normalized to 3.51 ng/μL DNA for PCR amplification. The V4 hypervariable region of the 16S rRNA gene was amplified using single-indexed universal primers (U515F/806R) with standard adapter sequences (Illumina Inc., San Diego, CA). The PCR program for amplification was: 98 °C (3:00) + [98 °C (0:15) + 50 °C (0:30) + 72 °C (0:30)] × 25 cycles + 72 °C (7:00; min:s). The V4 region of the 16S rRNA gene was selected for library generation because this region yields optimal community clustering [48]. The Illumina MiSeq platform (V2 chemistry with 2 × 250-bp paired-end reads) was used to sequence pooled amplicons [49]. The University of Missouri Informatics Research Core Facility binned, assembled, and annotated the DNA sequences. The FLASH software [50] was used for assembly. Sequences were trimmed, a minimum base quality of 31 was applied, and short sequences were removed. The de novo and reference-based chimera detection and removal was performed by using the Qiime v1.8 software [51]. The contiguous sequences were then assigned to operational taxonomic units (OTU). A minimum of 97% nucleotide identity was required for the de novo OTU clustering. The BLAST program [52] and SILVA SSURef database release 128 [53] was used for taxonomy assignment. The Qiime program also generated a table with the relative abundance of each OTU in each sample for comparative analysis.

### mRNA sequencing and data processing

Total RNA was extracted from endometrium using a Trizol-based method [54]. 260:280-nm absorbance ratios ranged from 1.91 to 2.03 (as determined by NanoDrop ND-1000 spectrophotometer; NanoDrop Technologies LLC). Each library was quantified by fluorimetery (Qubit quant-iT HS dsDNA reagent kit, Invitrogen). RNA quality numbers ranged from 6.8 to 9.2, and 28S:18S ratios ranged from 0.6 to 1.7 (as determined by the Fragment Analyzer instrument; Advanced Analytical Technologies, Inc). Fifty samples (26 at week 5 and 24 at week 9) were submitted for RNA library preparation using Illumina’s TruSeq mRNA stranded sample preparation kit at the University of Missouri DNA Core Facility. The libraries were sequenced using an Illumina NextSeq 500 sequencer to generate > 45 million 75-bp single-end reads per sample. The raw sequences (FASTQ) were subjected to FastQC (www.bioinformatics.babraham.ac.uk/projects/fastqc/) tool for checking sequence quality. The adapter sequences were removed by cutadapt [55]. The program fqtrim (https://ccb.jhu.edu/software/fqtrim/) was used to perform quality trimming (phred score > 30) by a sliding window scan (6 nucleotides), and remove reads shorter than 20 bp. Reads obtained from the quality control step were mapped to the bovine reference genome (UMD3.1) by using Hisat2 aligner [56]. The Ensembl gene annotation along with the alignment files were used in FeatureCounts tool [57] to quantify reads that mapped to each gene by using the sequences alignment files of each sample.

### Histological analysis of endometrium samples

Fixed endometrium was processed and sections were stained with hematoxylin–eosin at the Univeristy of Missouri Veterinary Medicine Diagnostic Laboratory. A Leica DM 4000B microscope (Buffalo Grove, IL) fit with a Leica DFC 450C camera was used to measure the diameter of individual lymphocytic foci (400X magnification). The diameter ranges used to classify the individual lymphocytic foci were: small (< 100 μm diameter), intermediate (100–250 μm diameter), and large (> 250 μm diameter). Inflammation was scored in the epithelium, superficial stroma, and deep stroma. The number of inflammatory cells (primarily neutrophils, macrophages and lymphocytes) was counted in ten random fields. Statistical analyses were based on the average number of inflammatory cells in epithelium, superficial stroma, and deep stroma.

### Blood metabolite and hormone analysis

Circulating glucose concentrations were determined enzymatically by the glucose oxidase method (Pointe Scientific Inc., Canton, MI) and circulating fatty acids concentrations were determined using a NEFA C kit (Wako Diagnostics, Richmond, VA) [58]. Circulating beta hydroxybutyrate was determined enzymatically using nicotinamide adenine dinucleotide (Sigma-Aldrich, St. Louis, MO) and 3-hydroxybutyrate dehydrogenase (Roche Diagnostics Co., Indianapolis, IN) [59]. Circulating insulin-like growth factor 1 (IGF1) [60] and P4 [61] concentrations were analyzed by validated radioimmunoassay. The intra- and inter-assay coefficients of variation were 9.1 and 9.9% for the P4 pools. The intra- and inter-assay coefficients of variation were 10.1 and 7.8% for the IGF1 pools.

### Data handling and statistical analysis

The 35 cows enrolled in the study were characterized as follows (Fig. 9). Week 5 cycling cows (CycW5; *n* = 10; 28.6%) had a CL present and circulating P4 concentrations ≥1.32 ng/mL on week 5. Week 5 non-cycling cows (NoCycW5; *n* = 19; 54.3%) had at least one follicle with a diameter greater than 11 mm present and no CL present on week 5 and circulating P4 concentrations below the detection limit of the assay (0.2 ng/mL) on week 1 to 5. Data from cows (*n* = 4; 11.4%) with circulating P4 concentrations ≥7.5 ng mL^− 1^ on week 4 but circulating P4 concentrations ≤0.5 ng mL^− 1^ on week 5 and either no CL (*n* = 2) or a CL with a large lacuna (n = 2) on week 5 were not included in the analysis because they were cycling but their uterus was not under P4 influence at the time of endometrial biopsy. Two cows (5.7%) with serious health complications unrelated to the study required euthanization. To be included in the statistical analysis, each cow needed microbiota data for week 1, 5, and 9, and transcriptome data for week 5 and 9. Of the 29 eligible cows, 14 did not meet these criteria due to the inability to perform a biopsy procedure or the unavailability of RNA of sufficient quality or quantity for sequencing. Fifteen cows (*n* = 7 CycW5 and *n* = 8 NoCycW5) with a complete set of microbiota data (week 1, 5, and 9) and transcriptome data (week 5 and 9) were retained for analysis. Power analysis calculated that with 15 cows, a gene-specific mean and dispersion and allowing different fold changes for each gene, there was 78% power to detect effects at an FDR of 4% [62].

Operational taxonomic units with an average relative abundance less than 1% on each week of sampling (1, 5, 9, and) were removed from the microbiota dataset prior to statistical analysis. Permutational multivariate ANOVA (PERMANOVA) of 1/4-root-transformed relative abundance microbiota data were implemented in PAST version 3.14 [63] to test for effects of week (1, 5, 9, and) and week 5 status (CycW5, NoCycW5) on microbial composition, using the Bray-Curtis similarity index, a measure of compositional similarity of the microbiota based on OTU abundance. Using PAST, principal components (PC) were generated from the week 1, 5, and 9 OTU data together and separately. Principal component analysis is a multivariate data analysis approach very suitable for studying bacterial communities. Its central aim is to reduce the dimensionality (the number of OTU) of the dataset while accounting for as much of the original variation as possible in the dataset. This aim is achieved by transforming to a new set of variables, the PC, that are linear combinations of the original variables (OTU), which are uncorrelated and are ordered so that the first few PC account for most of the variation in all the OTU [64]. In this manner, the OTU within each individual cow can be expressed as a series of PC (PC1, PC2, etc.) which are continuous numeric values (from negative to positive) which represent the overall composition of their microbiota and can be used in statistical analyses. Importantly the PC are orthogonal; meaning that they are independent and explain different sources of variation within the endometrial microbiota.

Transcriptome data were analyzed within the R statistical programming language (version 3.4.1) [65]. Counts per million for each transcript were calculated using the ‘DGElist’ function of the Bioconductor software package edgeR [66] and differential expression analysis was performed using the Bioconductor software package limma [67]. Preliminary model testing fitted each microbiota PC separately with week 5 ovarian cyclicity status to identify the important variables associated with differential gene expression. The final model for statistical analysis included the fixed effects of week 5 ovarian cyclicity status (CycW5 or NoCycW5), week (5 or 9), microbiota PC (WK1_PC1, WK1_PC2, WK5_PC1, WK5_PC2, WK9_PC1, WK9_PC2), and the interactions of week 5 ovarian cyclicity status x week postpartum and microbiota PC x week. Cow was included in the model as a random effect. The limma package applied empirical Bayes methods to compute moderated t-tests. Transcripts were deemed differentially expressed at *P ≤* 0.05 after adjustment for multiple testing using the Benjamini and Hochberg (BH) method.

Ingenuity Pathway Analysis (IPA; Qiagen, Redwood City, CA, www.qiagen.com/ingenuity) was used for the analysis of differentially expressed genes (DEG) derived from individual datasets. Transcripts were mapped to a single gene to create a new dataset for analysis that was subjected to Core analysis using the Ingenuity Knowledge Base. A ZS is a prediction of inhibition (< 0) or activation (> 0). Enriched canonical pathways that included metabolic and cell signaling pathways, upstream regulators, and biological functions were identified (*P* ≤ 0.01 and ZS ≥ |2|).

Regulatory effects analysis within IPA was used to identify the relationships between upstream regulators and biological functions. The default setting was used in the analysis meaning that upstream regulators were limited to genes, RNA, and proteins. There was no limit placed on the size of the network. The “consistency score” is a measure of the consistency and density of the network. A positive consistency score indicated a relevant regulator effects network.

A univariate mixed model analysis was performed in SAS 9.4 using PROC MIXED [68]. Data were assessed for normality and transformed if necessary. Energy-corrected milk production was calculated as 0.25 × milk yield (kg) + 12.2 × fat content (kg) + 7.7 × protein content (kg). Preliminary statistical analysis tested the effect of week 5 status (CycW5, NoCycW5) on microbiota PC 1 and 2 on week 1, 5, and 9, separately, and indicated these variables were not associated with each other. The number of 16S rRNA sequence reads, histology data, milk production, vaginal mucus score, rectal temperature, metabolic and reproductive hormones and metabolites were analyzed in a repeated measures analysis within an autoregressive covariance (AR1) structure. Week 5 status (CycW5, NoCycW5), week (1 to 20), microbiota PC (WK1_PC1, WK1_PC2, WK5_PC1, WK5_PC2, WK9_PC1, WK9_PC2), and the interaction of week 5 status x week postpartum were fitted as fixed effects with cow nested within week 5 status as a random effect. Effects were deemed significant if *P ≤* 0.05 after applying the Tukey adjustment to correct for multiple comparisons. Contrasts were written to compare variables of interest between week 1, 5, and 9 and between CycW5 and NoCycW5 cows. Solutions were requested to identify the parameter estimates for the effect of the microbiota PC. In a separate analysis, Wilcoxon tests were performed to test the effect on week 5 status on the relative abundance of OTU using PROC NPAR1WAY.

## Additional files

Additional file 1: **Table S1.** Relative abundance of operational taxonomic units in endometrium of postpartum dairy cows. **Table S2.** Endometrial genes differentially expressed at week 5 postpartum between cycling and non-cycling dairy cows. **Table S3.** Endometrial genes differentially expressed in dairy cows at week 5 postpartum in response to WK1_PC1 microbiota principal component. **Table S4.** Endometrial genes diffentially expressed in dairy cows at week 5 postpartum in response to WK1_PC2 microbiota principal component. **Table S5.** Endometrial genes differentially expressed in dairy cows at week 5 postpartum in response to WK5_PC1 microbiota principal component. **Table S6.** Endometrial genes differentially expressed in dairy cows at week 5 postpartum in response to WK5_PC2 microbiota principal component. **Table S7.** Endometrial genes differentially expressed between weeks 5 to 9 postpartum in dairy cows not-cycling at week 5 and cycling at week 9 (day 13 of the estrous cycle)**. Table S8.** Endometrial genes differentially expressed between weeks 5 to 9 postpartum in dairy cows cycling at week 5 and 9 (day 13 of the estrous cycle)**.Table_S9.** Endometrial genes differentially expressed at week 9 postpartum (day 13 of the estrous cycle) between dairy cows cycling and not-cycling at week 5 postpartum. **Table S10.** Endometrial genes differentially expresed in dairy cows at week 9 postpartum (day 13 of the estrous cycle) in response to WK1_PC2 microbiota principal component. **Table S11.** Endometrial genes differentially expressed in dairy cows at week 9 postpartum in response to WK9_PC2 microbiota principal component. **Table S12.** Endometrial genes differentially expressed in dairy cows at week 9 postpartum (day 13 of the estrous cycle) in response to WK1_PC1 microbiota principal component. **Table S13.** Endometrial genes differentially expressed in dairy cows at week 9 postpartum (day 13 of the estrous cycle) in response to WK5_PC1 microbiota principal component. **Table S14.** Endometrial genes differentially expressed in dairy cows at week 9 postpartum (day 13 of the estrous cycle) in response to WK5_PC2 microbiota principal component. **Table S15.** Endometrial genes differentially expressed in dairy cows at week 9 postpartum (day 13 of the estrous cycle) in response to WK9_PC1 microbiota principal component. (XLSX 1008 kb)

Additional file 2: **Figure S1.** Linear regression of 39 endometrial genes differentially expressed in lactating dairy cows between week 5 and week 9 postpartum in cows that were either cycling or non-cycling by week 5. **Figure S2.** Linear regression of 21 endometrial genes differentially expressed at week 5 and week 9 postpartum between lactating dairy cows that were either cycling or non-cycling at week 5. **Figure S3.** Linear regression of 27 endometrial genes differentially expressed in lactating dairy cows at week 5 and 9 postpartum in response to the endometrial microbiota at week 1 (WK1_PC1). (XLSX 174 kb)

## Abbreviations

AI: artificial insemination
CIDR: controlled internal drug release
CycW5: cows that resumed ovarian cyclicity by week 5 postpartum
DEG: differentially expressed gene
IGF1: insulin-like growth factor 1
IPA: Ingenuity Pathway Analysis
NoCycWk5: cows that resumed ovarian cyclicity by week 5 postpartum
OTU: operational taxonomic unit
P4: progesterone
PCA: principal component analysis
PGF_2α_: prostaglandin F_2α_
WK1_PC1: first principal component of week 1 endometrial microbiota
WK1_PC2: second principal component of week 1 endometrial microbiota
WK5_PC1: first principal component of week 5 endometrial microbiota
WK5_PC2: second principal component of week 5 endometrial microbiota
WK9_PC1: first principal component of week 9 endometrial microbiota
WK9_PC2: second principal component of week 9 endometrial microbiota
ZS: z-score
